# Percutaneous endoscopic lumbar partial laminectomy assisted by a new miniature parallel surgical robot system: a trial on a cadaveric specimen

**DOI:** 10.20452/wiitm.2025.17935

**Published:** 2025-03-24

**Authors:** Nan Su, Jiashen Shao, Gang Zhu, Yu Wang

**Affiliations:** Department of Orthopedic, Beijing Friendship Hospital, Capital Medical University, Beijing, China, China; Beijing Rossum Robot Co., Ltd, Beijing, China; School of Biological Science and Medical Engineering, Beihang University, Beijing, China; Beijing Advanced Innovation Center for Biomedical Engineering, Beihang University, Beijing, China

**Keywords:** cadaveric specimen, percutaneous lumbar laminectomy, preoperative planning, spinal endoscopy, surgical robot

## Abstract

**INTRODUCTION:**

Robot‑assisted surgery is becoming increasingly popular and its application is expanding to various spinal surgical procedures, including endoscopic spinal surgery.

**AIM:**

The aim of this study was to describe a novel small parallel orthopedic surgical robot and evaluate its feasibility in assisting surgeons during percutaneous lumbar laminectomy on cadaveric specimens.

**MATERIALS AND METHODS:**

The authors of the study developed a new orthopedic surgical navigation system (R‑Pharos, Rossum Robot Co., Ltd, Beijing, China), consisting of a navigation cart and a hybrid serial‑parallel bedside robotic arm. The system is equipped with interactive software for selecting and planning the percutaneous lumbar laminectomy target and path. A cadaveric specimen was selected for a right‑side partial laminectomy at L4. During the procedure, the surgeon used the robotic arm to guide the saw to the target lamina and perform the percutaneous resection. Postoperative cone beam computed tomography (CBCT) and endoscopic assessments were used to confirm the resection outcome.

**RESULTS:**

After optimizing the precision of the small parallel orthopedic surgical robot to 1 mm, it was shown to meet the navigational requirements for percutaneous lumbar laminectomy. The surgeon utilized the interactive software to design the resection range and path for the right L4 lamina which was successfully resected, as confirmed by endoscopic observation. A postoperative CBCT scan revealed that the resection area precisely matched the preoperative design.

**CONCLUSIONS:**

This study demonstrated that the small parallel orthopedic surgical robot was capable of preoperatively planning the lamina resection area and could assist the surgeon in performing percutaneous lumbar laminectomy with high navigational precision.

## INTRODUCTION

Lumbar spinal stenosis (LSS) is a type of degenerative lumbar disease that leads to neurogenic claudication and is often accompanied by lower back pain.^1^ Since the early 1980s, when spinal endoscopy started being used to treat lumbar disc disease,^2^ this field has been developing rapidly. Unilateral laminectomy for bilateral decompression (ULBD) surgery is a common minimally invasive lumbar decompression procedure performed using spinal endoscopy to treat LSS. Compared with traditional open lumbar decompression surgery, its advantages include a smaller incision, less damage to soft tissues and paraspinal muscles, less bleeding, shorter hospital stays, and faster patient recovery. However, performing ULBD with spinal endoscopy requires high surgical skills. The key to successful surgery is the correct placement of the working channel of the spinal endoscope at the target decompression site, which helps shorten the operation time and prevents in‑ traoperative complications. This step requires significant surgical experience, making the ULBD learning curve quite steep.^3^

**FIGURE 1:**
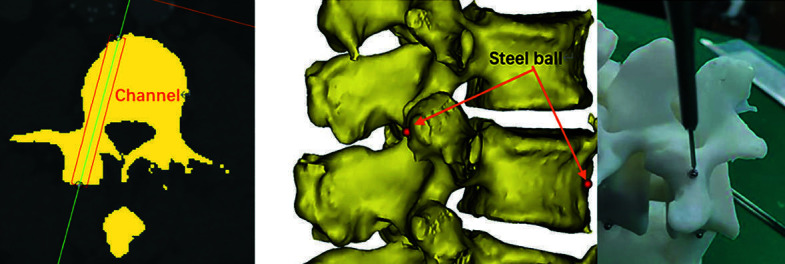
Precision test procedure. The channel indicates as pedicle screw was drawn on computed tomography (CT) scan imaging (A). Two ends of the channel: the foci of the channel and the facet, and the channel and the anterior edge of vertebral body, were marked (B). We made a 3‑dimensional printing model according to the CT data and placed the steel balls on it. A joint‑type 3‑dimentional coordinate measuring machine was used to measure the straight‑line distance from the steal balls to the central axis of the gauge (C).

In the past decade, robot‑assisted spine surgery has achieved significant success. Robotic systems can provide precise positioning guidance and execute preoperative plans. Numerous studies have shown that robot‑assisted pedicle screw placement improves accuracy and reduces intraoperative radiation exposure compared with traditional manual techniques.^4^^,^^5^^,^^6^ By adjusting software and upgrading the design of the manipulator, orthopedic robots can also assist in lumbar decompression surgery, particularly in guiding endoscopic lumbar laminotomy. With robotic assistance and other instruments, surgeons can perform bone decompression operations such as laminectomy and partial resection of the facet. Subsequently, an endoscope can be inserted to carry out soft tissue decompression, including, among others, flavectomy or discectomy. This approach optimizes the surgical process of endoscopic lumbar spinal decompression.

We designed a new miniature parallel surgical robot system. Using this robot, we assisted surgeons in performing partial lumbar laminectomy through percutaneous channels on cadaveric specimens, aiming to explore its potential use in endoscopic lumbar laminectomy.

## AIM

The aim of this study was to describe a novel small parallel orthopedic surgical robot and evaluate its feasibility in assisting surgeons during percutaneous lumbar laminectomy on cadaveric specimens.

## MATERIALS AND METHODS

### Robot design

In this study, we used a new orthopedic surgical navigation system (R‑Pharos, Rossum Robot Co., Ltd, Beijing, China). The system consists of 2 parts: the navigation cart, which includes surgical tool navigation system and a human‑machine interface (HMI), and the manipulator. The navigation cart is used for patient image registration. The HMI allows surgeons to set the decompression channel on the image. The manipulator is partially used to execute accurate movements. The manipulator used in this study is a series‑parallel hybrid manipulator bedside connection system. The weight of the manipulator system is 12.5 kg, and its arm span is 800 mm. It is directly fixed to the surgical bed via a bedside rail.

The series part is a custom‑made passive control system with 5 joints and 9 degrees of freedom, capable of handling a 15 kg load. It can be manually dragged and locked in any position. The parallel part is a miniaturized Stewart platform that can provide small‑range, 6‑degree‑of‑freedom movements within a 30 mm diameter cylinder. The upper part of the Stewart platform features a special joint that connects either the guide or the phantom to the optical position sensor.

At the end of the parallel structure, there is a display that shows the direction of the normal plane of the guide. The display moves along with the parallel structure. This screen connects to a computer via Bluetooth, allowing real‑time monitoring of the projected coordinates of the planned screw head and tail on the normal plane of the guide. By moving the end structure and aligning the screw head and tail on the screen, the rough desired position is achieved. Once the operator locks the position, the parallel structure ensures precise placement. Compared with a fixed monitor, this movable display bet‑ ter reflects the positional relationship between the end guide and the spatial screw channel, mak‑ ing it easier for the operator to achieve the desired rough position.

During the procedure, we first securely connected the manipulator to the surgical bed and attached a 3‑dimensional phantom to the end of the manipulator. Next, we installed a track‑ er at the posterior inferior iliac spine to serve as the global reference point. By releasing the passive control system, the 3‑dimensional phantom was positioned above the specimen’s spine. We then used cone beam computed tomography (CBCT) scanning to complete the registration.

**TABLE 1:** Results of the precision test in the first lumbar model

Entry point of each level	Precision, mm	Removal point of each level	Precision, mm
L1	0.654	L1	1.049
L2	0.518	L2	1.002
L3	0.879	L3	1.000
L4	0.560	L4	1.130
L5	1.080	L5	1.050
R1	0.880	R1	1.068
R2	0.631	R2	0.946
R3	0.688	R3	0.580
R4	0.660	R4	0.750
R5	0.880	R5	0.650

Abbreviations: L, left; R, right

**TABLE 2:** Results of the precision test in the second lumbar model

Entry point of each level	Precision, mm	Removal point of each level	Precision, mm
L1	0.528	L1	0.794
L2	0.530	L2	0.680
L3	0.270	L3	0.320
L4	0.770	L4	0.970
L5	0.610	L5	0.760
R1	0.770	R1	0.970
R2	1.110	R2	0.830
R3	1.030	R3	1.030
R4	0.210	R4	0.520
R5	1.010	R5	1.160

Abbreviations: see TABLE 1

The surgeons planned the desired screw tra‑ jectory on the registration image of the spine. After planning, we removed the 3‑dimensional phantom and installed the guide. The passive control system moved the guide to the position close to the target vertebra and locked it in place. At this point, the platform precisely aligned with the planned screw trajectory. The surgeons could then insert the gauge along the guide’s direction. Once the gauge reached the bone surface, the sur‑ geons drilled the K‑wire into the vertebral body and completed pedicle screw placement along the K‑wire.

### Verification of precision 

CT models of the lumbar spine and pelvis from 2 patients were used for 3‑dimensional reconstruction and 3‑dimensional printing. The surgeon designed the trajectory of the pedicle screw and drew a channel from the facet to the anterior edge of vertebral body. The 2 end points of this channel (entry point and removal point) were marked. To verify the precision of the system, we installed a steel ball in each point for each vertebral segment and a tracker at the posterior inferior iliac spine in the 3‑dimensional printing model. During the precision test, we found the position of the steel ball in the CT 3‑dimensional reconstruction model and moved the robotic arm to the ideal position. Then, we used a joint‑type 3‑dimensional coordinate measuring machine to find the central axis coordi‑ nates of the gauge and the coordinates of the steel balls, respectively. The deviation was calculated as the straight‑line distance from the steal balls to the central axis of the gauge, which was represented as a system error. This procedure is illustrated in FIGURE 1. As there were lumbar CT scan data from 2 patients, a total of 20 pedicle channels (L1 to L5, bilaterally) were recorded and the pre‑ cision was measured one by one. The results of the precision test are presented in TABLE 1 and TABLE 2.

### Statistical analysis

Data analysis was conducted using the R software (The R Foundation for Statistical Computing, Vienna, Austria). The preci‑ sion of the entry and removal points is preserved to 3 significant digits.

### Ethics statement

The experimental protocol was reviewed and approved by the Ethics Committee of the Beijing Friendship Hospital (2022KY087).

## RESULTS

### Cadaveric specimen simulation surgery 

**FIGURE 2:**
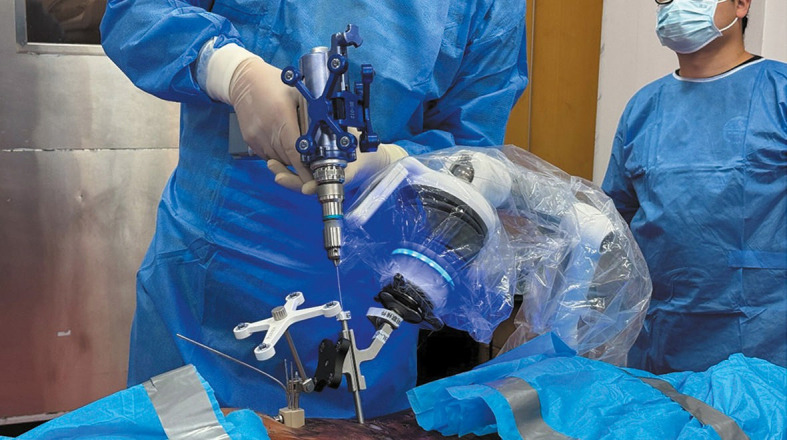
A novel orthopedic surgical navigation system (R‑Pharos, Rossum Robot Co., Ltd, Beijing, China) assists during pedicle screw placement.

Previous studies and cadaver research have proven that the studied spinal surgical robot could assist during pedicle screw placement with high accuracy (FIGURE 1 and FIGURE 2). Moreover, we explored the use of this surgical robot to navigate other spinal surgical procedures, such as partial lumbar laminectomy. Below, we describe the simula‑ tion of the robot‑assisted partial lumbar laminectomy process on a cadaveric specimen.

**FIGURE 3:**
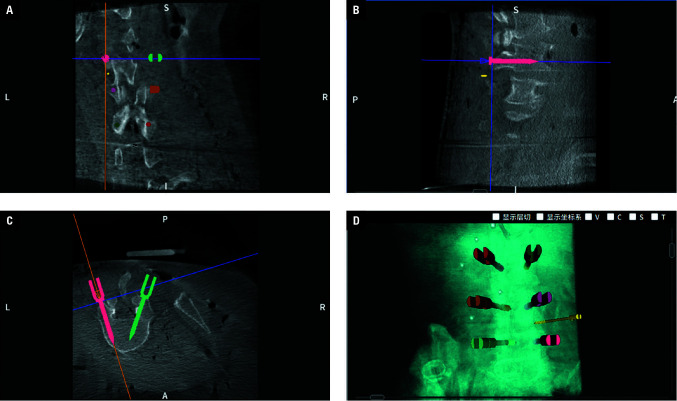
The 2‑dimensional (A–C) and 3‑dimensional (D) reconstruction models of the vertebrae obtained by cone beam computed tomography scanning of the specimens

Using a human cadaveric specimen, we performed robot‑assisted percutaneous lumbar partial laminectomy, targeting the right lamina of L4. The specimen was placed in the prone position on the surgical table, with the R‑Pharos miniature parallel surgical robot positioned on its right side and fixed to the surgical bedframe. After using fluoroscopy to locate L3–L5, the surgeon made a longitudinal incision on the surface of the L3 spinous process. The skin, subcutaneous tissue, and deep fascia were incised to expose the sur‑ face of the L3 spinous process where we placed a tracker and ensured that it could be detected by the network device interface stereo camera (optical position sensor). Through CBCT scanning of L3–L5 of the specimen, we obtained both 2‑ and 3‑dimensional reconstructions of the vertebrae. The reconstruction model is shown in FIGURE 3.

**FIGURE 4:**
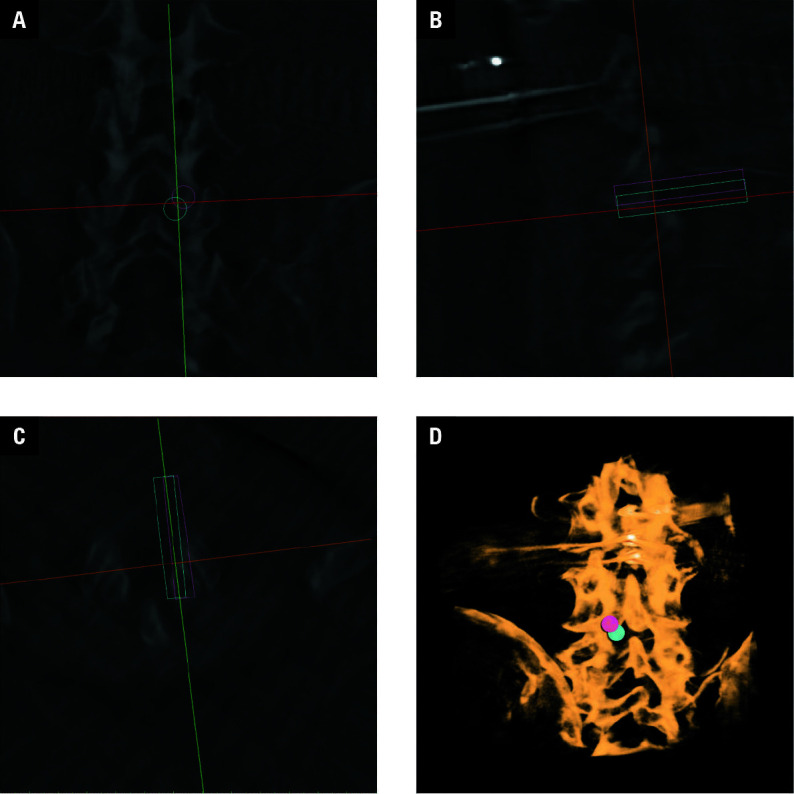
Two lamina target sites were designed by the 2‑ and 3‑dimensional reconstruction spinal models. The figure displays coronal (A), sagittal (B), cross‑sectional (C), and 3‑dimensional reconstructed images (D) of the area where the target is located. Two cylinders with a 0.8 cm diameter, which represented the trephine, were generated. The first target site was located at the junction of the lower edge of the L4 lamina and the inferior articular process of L4. The second target site was on the rostral side of the first target site, overlapping partially.

Using this model, we could plan the target laminectomy site and the operation pathway. Since the surgical tool for the laminectomy was a trephine with a diameter of 0.8 cm, we used soft‑ ware to generate a cylinder of the same diameter and placed it in the spinal model. In the coronal section, we moved the cylinder to the junction of the lower edge of the L4 lamina and the inferior articular process of L4, which was the target lam‑ inectomy site. Then, we adjusted the position of the cylinder in the sagittal plane, making it per‑ pendicular to the lamina. By reverse stretching of the cylinder, we could determine the position of the skin incision point, which was the intersection of the cylinder with the skin. The 3‑dimensional coordinates of the skin incision and the target lamina point were recorded, with the skin incision as the entry point and the target lamina point as the exit point. The design of the target laminec‑ tomy site is shown in FIGURE 4.

**FIGURE 5:**
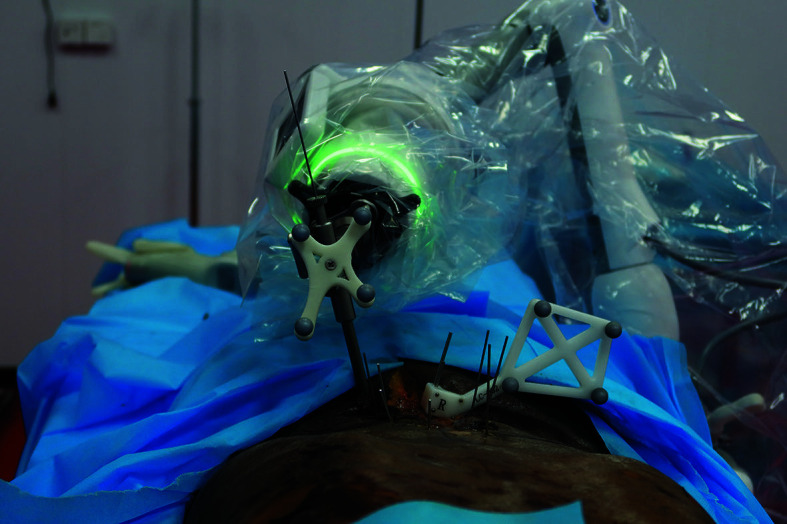
The surgeon used the trephine to remove a part of the lamina with the R‑Pharos miniature parallel surgical robot assistance.

Afterwards, the surgeon moved the manipula‑ tor near the skin incision. Guided by the display on the manipulator, the surgeon adjusted the manipulator’s position until the indicator light turned green, and then locked it in place. The remote platform of the manipulator was further activated to adjust the precision between the en‑ try and exit points to within 1 mm. The surgeon then inserted the trephine along the guide, ensuring the entry point was where the trephine contacted the skin. The skin, subcutaneous tis‑ sue, and deep fascia were cut. As the trephine approached the bone surface, the surgeon con‑ firmed that the precision was still within 1 mm, used the trephine to remove a part of the lami‑ na, and then dismounted the trephine. The sur‑ gical procedure and the excised bone specimen of the lamina are shown in FIGURE 5.

Reusing the same spinal reconstruction model, we could design and plan the second laminectomy target site on the rostral side of the first tar‑ get site. To avoid leaving residual lamina when using the trephine, we needed to partially overlap the second laminectomy target site with the first one to ensure that the distance between the 2 cen‑ ters was less than 1.5 cm. The second target site is illustrated in FIGURE 4.

Since no additional incisions were needed, the original incision served as the entry point, and the second target site as the exit point, with the line connecting them representing the trephine’s trajectory. We then used software to re‑ cord the coordinates of each point mentioned above. The manipulator platform was activated to ensure that the precision between the entry and exit points was always within 1 mm. The surgeon then reinserted the trephine through the origi‑ nal incision to the second target on the lamina surface, excised part of the lamina, and removed the trephine. The precision of the entry and exit points is detailed in TABLES 1 and 2.

The spinal endoscope was inserted through the skin entry point. Another CBCT scan of L3–L5 of the cadaveric specimen was performed, allow‑ ing for 2‑ and 3‑dimensional reconstructions. Through these reconstructions, we could observe

## DISCUSSION

As endoscopic spinal surgery is a widely used procedure, an increasing number of surgeons employ this technology to perform lumbar laminectomy for LSS. Unlike simple endoscopic discectomy, it is still a challenge for surgeons to use endoscopy to perform lumbar partial laminectomy, even ULBD. Compared with lumbar disc herniation (LDH) patients, individuals with LSS are elderly and suffer from more severe lum‑ bar degeneration. The lumbar anatomical land‑ marks may be ambiguous as the osteophyte and hypertrophy ligaments interfere. Surgeons need more training and experience to perform the en‑ doscopic lumbar decompression surgery for LSS than in the case of LDH.^7^ The latest study shows that surgeons can overcome the learning curve after performing 35 surgeries, which is when op‑ eration time and radiation exposure decrease sig‑ nificantly.^8^ Compared with traditional open lum‑ bar decompression surgery, percutaneous endo‑ scopic lumbar laminectomy (PELS) is significantly less invasive, with smaller incision, less bleeding, and better lumbar posterior muscles and ligaments protection, but it is not completely safe. Even for experienced surgeons, the complication rate for PELS is still 6%–12%, including, among others, dual tear, nerve root injury, hematoma, or insufficient decompression.^8^^,^^9^^,^^10^

To help surgeons overcome the learning curve, it is essential to simplify PELS procedures by enhancing positioning accuracy, reducing surgery time, and minimizing intraoperative radiation exposure. As a result, numerous new technologies have been integrated into PELS surgeries. One no‑ table technology is the navigation system which provides real‑time positioning information, enabling surgeons to precisely locate the position and depth of surgical instruments.^11^ Research has demonstrated that the use of O‑arms or other navigation systems facilitates accurate punc‑ tures and effective surgical path guidance during PELS procedures.^12^^,^^13^ However, it does have certain drawbacks. Surgeons must concentrate on the navigation interface, making it challenging to simultaneously monitor the surgical image through the spinal endoscope. Consequently, using the navigation system requires surgeons to possess strong hand‑eye coordination skills.

In recent years, the advancement of surgical robots has led to significant achievements in spinal surgery. Many studies have demonstrated that robot‑assisted surgery enhances the accuracy of pedicle screw placement and reduces intraopera‑ tive radiation exposure, particularly in minimally invasive lumbar surgeries.^4^^,^^5^^,^^6^,^14^ Compared with traditional navigation systems, robot‑assisted surgery allows surgeons to preplan the proce‑ dure, eliminating the need for real‑time monitoring of the robotic interface. Based on the success of orthopedic surgical robots in pedicle screw placement, we believe their application can be extended to assist with lumbar decompression surgeries under spinal endoscopy. Therefore, we developed this robotic system and applied it to assist in lumbar laminectomy.

**FIGURE 6:**
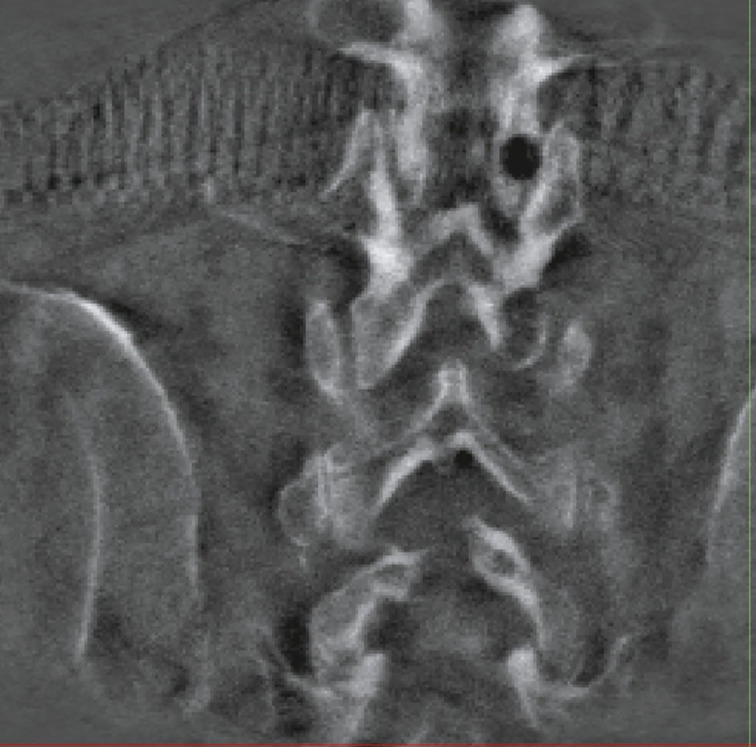
The partial bones of lamina (arrow) were precisely removed, as confirmed by the postoperative cone beam computed tomography scan and imaging reconstruction.

Using the patient’s lumbar CT data, surgeons can preoperatively select the target lamina resection area, design the optimal puncture path, and determine the corresponding skin incision location. In our initial trials, we successfully performed partial lumbar laminectomy on cadaver specimens with robotic assistance. By compar‑ ing preoperative and postoperative lumbar CT scans, we verified that the intraoperative lamina resection closely matched the preoperatively designed target, demonstrating high accuracy of this approach. With preoperative planning and robotic‑assisted positioning, surgeons can achieve precise lamina resection, minimize unnecessary bone removal, and avoid postoperative iatrogenic spinal instability. Additionally, robotic assistance ensures that the puncture process and subsequent partial lumbar laminectomy are not compromised by abnormal anatomical landmarks or other intraoperative visual impediments, such as bleeding or bone debris from drilling. It also mitigates the impact of the surgeon’s lack of experience. With robotic assistance, surgeons can minimize damage to critical anatomical struc‑ tures, shorten surgery time, and reduce intraop‑ erative radiation exposure.

There are various types of surgical instruments for endoscopic lumbar laminectomy, such as drills, piezoelectric osteotome, or trephines. Li et al^15^ designed a novel spinal robot with a piezoelectric osteotome and force sensor. The robot to performed laminectomy autonomously. We believe, however, that trephines are particularly well‑suited for orthopedic surgical robots because they can be easily gripped and guided by the manipulator. Unlike high‑speed drills or piezoelec‑ tric osteotome, trephines perform circular rotational bone removal at a relatively low speed, making them easier forthe manipulator to con‑ trol. This allows surgeons to perform bone resections accurately. Furthermore, based on preoperative lumbar CT images, surgeons can determine the thickness of the target lamina for resection. Once the trephine is activated for bone removal, the manipulator can automatically limit its depth according to the lamina thickness. The trephine features a depth gauge, allowing surgeons to ob‑ serve the gauge markings through the endoscope and determine how deep the trephine has pene‑ trated the bone. This dual‑check system ensures safety during lamina resection. With a diameter of only 0.8 cm, the trephine allows surgeons to avoid unnecessary bone removal, preserve the in‑ tegrity of the facet joints, and reduce the risk of postoperative iatrogenic lumbar instability. Given these advantages, we selected trephines for lami‑ na resection and cadaveric specimen experiments. For this study, we selected a new type of min‑ iature parallel surgical robot and proposed an in‑ novative serial‑parallel hybrid manipulator sys‑ tem. This system is smaller and lighter than com‑ mon manipulator platforms such as Tirobot,16,17 Mazor X,18 and Globus,19 providing a spatial ad‑ vantage in the operating room. Since the manip‑ ulator is fixed to the surgical bed, there is no ob‑ struction from the platform below the bed, mak‑ ing it more convenient for the C‑arm to capture additional fluoroscopic verification positions if needed. The small size of the manipulator also minimizes interference with the surgeon’s oper‑ ating area. Miniature parallel robots offer high‑ er rigidity compared with serial robots and ex‑ hibit lower inertia during movement, allowing them to respond to the patient’s breathing with smaller movements. This feature provides unique advantages for future applications, for example, in respiratory monitoring. However, as the robot is directly connected to the surgical bed, the bed needs to be more rigid. The overall rigidity of this system is slightly lower than that of platform‑based manipulators. To ensure stability, surgeons need to press the gauge against the bone surface before drilling.

In the future, robot‑assisted lumbar laminectomy could bring significant transformative changes. It allows the manipulator to guide the direction and orientation of the surgical tool (such as the trephine) while simultaneously limiting its depth. In theory, when working with this method, the surgeons can even use the trephine to perform percutaneous laminectomy with robot‑ ic assistance. In this approach, they do not need to insert the endoscope and rely on its imaging. Once the bone resection is completed, the spinal endoscope can be used for further procedures, such as ligamentum flavum resection and discectomy, thus optimizing the process of spinal endoscopic surgery.

This study has some limitations. First of all, we simulated PELS surgery using a cadaveric specimen. In cadaveric specimens, there is no interference in the surgical field caused by bleeding or muscle tension affecting the precision of the manipulator’s movements. Secondly, the CBCT scan of the lumbar region does not provide a high‑resolution image for spinal reconstruction. Finally, robot‑assisted spinal endoscopic surgery requires an additional skin incision to expose the spinous process and place the tracker. In fu‑ ture studies, we need to further adjust the design of the surgical robot to meet the requirements for assisting lumbar decompression surgery un‑ der spinal endoscopy.

## CONCLUSIONS

Following precision optimization to 1 mm, the parallel orthopedic robotic system demonstrated compliance with navigational standards for percutaneous lumbar laminectomy. Utilizing interactive planning software, the right L4 lamina resection path was executed successfully, with endoscopic and CBCT confirmation of complete consistency between intraoperative outcomes and the preoperative design. This validates the system’s capability for high‑precision preoperative planning and intraoperative navigation in laminectomy procedures.
